# Methods and Guidelines for Measurement of Glucagon in Plasma

**DOI:** 10.3390/ijms20215416

**Published:** 2019-10-30

**Authors:** Jens J. Holst, Nicolai J. Wewer Albrechtsen

**Affiliations:** 1 Department of Biomedical Sciences, Faculty of Health and Medical Sciences, University of Copenhagen, 2200 Copenhagen, Denmark; nicolai.albrechtsen@sund.ku.dk; 2 Novo Nordisk Foundation Center for Basic Metabolic Research, Faculty of Health and Medical Sciences, University of Copenhagen, 2200 Copenhagen, Denmark; 3 Department of Clinical Biochemistry, Rigshospitalet, 2100 Copenhagen, Denmark; 4 Novo Nordisk Foundation Center for Protein Research, Faculty of Health and Medical Sciences, University of Copenhagen, 2200 Copenhagen, Denmark

**Keywords:** alpha cell, amino acids, diabetes, glucose, hyperglucagonemia

## Abstract

Glucagon circulates in concentrations in the low picomolar range, which is demanding regarding the sensitivity of the methods for quantification applied. In addition, the differential and tissue specific proteolytic processing of the glucagon precursor and the presence in of several glucagon-like sequences, not only in the precursor of glucagon, but also in a number of other peptides of the glucagon-secretin family of peptides, put special demands on the specificity of the assays. Finally, experience has shown that unspecific interference of plasma components has presented additional problems. All of these problems have resulted in a lot of diverging results concerning measured and reported glucagon responses in both humans and experimental animals that have and still are causing considerable debate and controversy. There is very solid evidence that glucagon is an important hormone in human and mammalian metabolism, but its precise physiological role in glucose and lipid metabolism and in metabolic disease has been difficult to establish, not least because of these difficulties. It was our purpose with this review to discuss the methods of glucagon quantification and discuss pitfalls and sources of error. We also reviewed some of the dogmas regarding glucagon secretion in the light of the methodological difficulties.

## 1. Introduction

Because of the low concentrations of circulating glucagon, direct measurements in plasma had to await the development of sufficiently sensitive techniques, and the advent of the radio-immunoassays allowed Roger Unger in Dallas, Texas, who was in close contact with Berson and Yalow, to build on their experience from the development of the insulin assay [1], enabling him (and Eisentraut) to publish the first glucagon assay, almost simultaneously with the insulin assay, in 1959 [2]. A full publication appeared in 1961 [3] and the assay was reported to have a detection limit of 50 pg/mL (16–17 pmol/L)- sensational for the time, but not quite enough to reliably measure physiological levels in humans. In the following years, Unger and the Unger lab made numerous important contributions to our knowledge of glucagon’s physiology, including the importance of glucose for its secretion [4,5,6], the effect of starvation [5], diagnosis of glucagon-secreting tumors [7] and the importance of amino acids for its secretion [8]. Assay improvements along the way made it possible to study meal response in both healthy and in individuals with type 2 diabetes, resulting in the citation classic by Müller et al. from 1970 [9], showing suppression of glucagon by carbohydrates but lack of suppression and hypersecretion of glucagon in patients with T2DM. The group was subsequently fortunate enough to develop a particularly sensitive and specific antiserum from rabbit 30 K with improved sensitivity and specificity [10], and the glucagon assay based on this antiserum, which was commercialized, dominated the field of glucagon research for decades. Eventually, other assays with similar or improved specificity and sensitivity were developed.

## 2. The Specificity of the Glucagon Assays

What were the particular specificity problems of the glucagon radioimmunoassays? Early work from Unger’s laboratory indicated that there was glucagon immunoreactivity in the gut [11,12], and work by Valverde from Unger’s group [13] and also from Buchanan in North Ireland [14], as well as Holst et al. in Copenhagen [15] indicated that there were glucagon-like substances secreted from the gut; mainly two components, differing in size both of which differed significantly from pancreatic glucagon. Antisera were developed that did not react with these intestinal moieties at all [10,16,17], whereas others appeared to react equally with the two forms intestinal forms and with glucagon, although all antisera were raised against glucagon. The intestinal molecules were called gut-GLI (gut glucagon-like immunoreactivity) or enteroglucagon [18]. Research from several groups finally revealed that both intestinal moieties contained the entire glucagon sequence [19]. After isolation and sequencing, the larger of the two molecules, which was designated glicentin (“GLI” for glucagon-like immunoreactivity, “cent” because Sundby, who first isolated the compound, thought it contained 100 amino acids [20], “-in” to make it sound hormone-like), was shown [21] to consist of 69 amino acids, of which glucagon occupies positions 33–61. The smaller one, of 37 amino acids with glucagon occupying to first 29 amino acids [22,23], was named oxyntomodulin by Bataille, who had found inhibitory activity of this compound on acid secreting oxyntic glands) in experimental animals [24].

**Figure 1:**
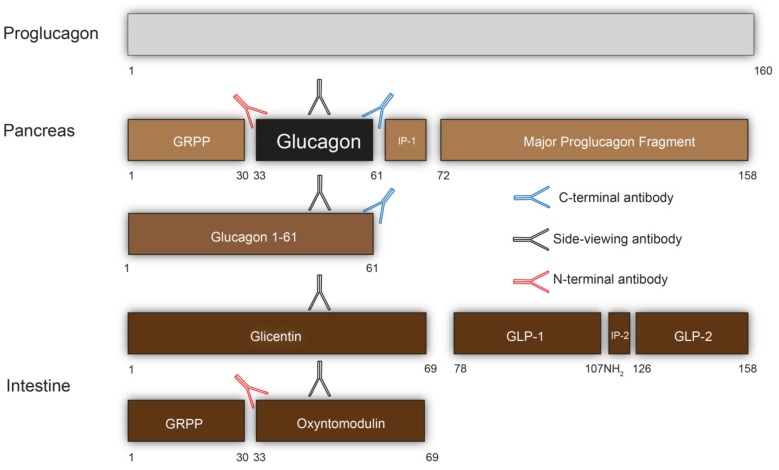
Proglucagon processing and measurement of glucagon by antibodies. The numbers refer to the numbering of the residues in proglucagon. Glucagon 1–61 may also be formed in the pancreas.

In further research, the peptide occupying positions 1–30 of glicentin (GRPP, glicentin-related pancreatic polypeptide) was isolated also from the pancreas and demonstrated to be released in parallel with glucagon [25], and intact glicentin was also found in pancreatic extracts [26]. Therefore, it was proposed that glicentin was at least a fragment of proglucagon, the glucagon precursor [25]. Proglucagon would be formed in both the intestinal mucosa and in the pancreas, but would undergo differential post-translational processing, leading to formation of glicentin and oxyntomodulin (but no glucagon) in the gut, and GRPP, glucagon, and a small C-terminal fragment in the pancreas. Upon cloning of the gene encoding proglucagon [27,28], this concept was found to be correct, but the proglucagon sequence also revealed the presence of two additional glucagon-related sequences in the remaining part of the precursor, henceforth designated the glucagon-like peptides-1 and -2. As was subsequently demonstrated, this part of the glucagon precursor, the so-called major proglucagon fragment (residues no 72–160), also undergoes differential tissue specific processing, releasing the two glucagon-like peptides from the gut, whereas it is secreted mainly as a single molecular entity from the pancreas [29,30]. Looking at the proglucagon processing (Figure 1), is becomes clear that the glucagon sequence is present in several molecular entities, which all may be present in the circulation and in the various tissues [31].

From the pancreas: the fully processed 29-amino acid glucagon peptide. In addition, there is some formation of proglucagon 1-61, comprising the glucagon sequence + GRPP (+ two basic residues removed during proteolytic processing) [32]. Under normal circumstances, proglucagon 1–160 (or maybe 1–158) is not known to be secreted, but the product may be difficult to recover during plasma sample processing and may be secreted in individuals with mutations of the prohormone convertases [33].

From the gut [34]: Glicentin, proglucagon 1–69. Glicentin is thought to have a rather prolonged half-life compared to the other proglucagon-derived peptides but is otherwise secreted in near equimolar amounts with GLP-1 and GLP-2. Oxyntomodulin is also secreted, usually in a ratio of 1:2 or 1:3 compared to glicentin. It is usually thought of as a processing product of glicentin, and it is stored in the L-cells in the same ratio as it is secreted [35]. Glucagon itself has also been found to be produced in the gut under certain circumstances, usually in small amounts [36] (see below).

## 3. The Glucagon Radioimmunoassay

The essential ingredients in a radioimmunoassay are [37]:

(1) A pure, correct standard. This is uncomplicated for a short peptide such as glucagon.

(2) A radiolabeled ligand. Unlike earlier, 125-I is generally used because of its safety and reasonable half-life. The iodine is incorporated by oxidation into a tyrosine residue of the molecule, and the essential maneuver is to ensure mono-iodination (better stability) and high specific activity, i.e., lack of admixture of un-labeled peptide. 131-I was previously used for glucagon radioimmunoassays, but is more sensitive for instability (e.g., decay catastrophe) compared to 125-I. Addition of aprotinin (trasylol) was previously important to reduce protease-dependent degradation of the tracer but may not be necessary for 125-I. Today, it is possible to purchase mono-iodinated glucagon tracer with high specific activity, but the tracer quality is always an important and potentially varying factor;

(3) An antibody with suitable affinity and specificity. High affinity is essential for the sensitivity. With appropriate immunization procedures it is possible to generate antibodies of very high affinity, and although such antisera are by nature polyclonal and have varying affinities, presence of even a very small fraction of antibodies with high affinity will suffice to establish a sensitive assay, because only this little fraction of all of the clones will be able to participate in ligand binding when the reagents are diluted to fit the concentrations of glucagon in plasma. Some of these clones may also have the desired specificity, but this always needs to be established by careful specificity testing.

How do we select antibodies with an appropriate specificity? Upon immunization with full length glucagon, a mid-region of the molecule usually shows up as antigenic determinant [38]. Such antibodies against a mid-region will measure all moieties containing the glucagon sequence, including those from the gut. These antibodies may be designated cross-reacting. Antibodies against the N-terminus of glucagon, which can be generated by immunization with N-terminal fragments of the molecule, will necessarily cross-react with oxyntomodulin and therefore be useless in a radioimmunoassay. Antibodies against the C-terminus, on the other hand, will cross-react exclusively with glucagon from the pancreas and with proglucagon 1–61, which is secreted in parallel with glucagon itself, and also has some glucagon bioactivity [32]. It is normally present in very small amounts, except in patients with renal disease, where this component may be the dominating circulating moiety. If glucagon also is secreted from the gut, this will of course also contribute. The famous antiserum, 30k, from the Unger lab, is a C-terminal antibody, and thus, is the antiserum 4305 used in the authors’ laboratory for many years. Indeed, most of our knowledge about glucagon physiology is obtained with such C-terminal antibodies, sometimes designated specific antibodies. The cross-reacting antibodies, on the other hand, measure glicentin and oxyntomodulin, and by subtracting the results obtained with a C-terminal assay from those obtained with a cross reacting assay, one may obtain a measure of the enteroglucagons (glicentin and oxyntomodulin) [39]. The strongest stimulus for the secretion of the enteroglucagons is luminal carbohydrates [40], and if carbohydrates in a meal are the predominating component, pancreatic secretion will be normally be suppressed, and the measured concentration will therefore mainly reflect the contribution of the enteroglucagons. Unfortunately, it has happened many times in the literature that investigators have not been aware of these differences, which means that many reported results are inaccurate. Indeed, unless specific reference is made to the specificity of the assay, reported findings regarding glucagon secretion should not be considered accurate.

### 3.1. Unspecific Interference

Whether the binding region of glucagon antibodies shows cross-reaction with plasma proteins is unknown, but it is a fact that most (virtually all) glucagon antibodies react unspecifically with proteins in plasma, causing a matrix effect. This can be quite pronounced and is therefore best avoided. It has been tried to use plasma from pancreatectomized individuals to correct for this error [41], but these patients may actually secrete glucagon from the gut (see below), causing misleading results. In the authors’ laboratory, we have always used some form of sample pretreatment, typically ethanol extraction (70–75% vol/vol final) [16]. The recovery after extraction is not 100% (rather around 70%), but is rather constant, and effectively removes the interference. Using this procedure and high affinity C-terminal antisera, the plasma concentration of glucagon typically falls from 7–10 mol/L in the fasting state down to around 1 pmol/L [42], consistent with the near complete cessation of glucagon secretion from isolated perfused pancreas preparation exposed to high glucose concentrations. The problem with nonspecific interference (noted fx with the 30 K assay as well as the previous Unger assays and with most commercial assays) is that the high baseline concentration is not only inaccurate but also to some extent blurs dynamic responses [42,43].

### 3.2. Other Important Factors

The assays are usually performed in conventional buffers, but because glucagon in dilution (like many peptides) sticks to glassware and plastics, some carrier, usually albumin, must be added. The quality of the albumin is critical, because many albumin preparations are contaminated with enzymes that may attack particularly the tracer. Today pure, recombinant albumin is available. Additionally, the method of separation is critical, and all components must be checked for interference in the assay. The same applies to the employed glassware.

## 4. The Enzyme-Linked Immunosorbent Assay, ELISA

A sandwich ELISA is the ideal solution to the specificity problem of the glucagon assay. For this, a pair of antisera are required that are “terminal wrapping”, meaning that their binding site is the free terminus of the molecule, such that any modification of the terminus, prolongation or truncation, leads to complete loss of binding to the antibody. As discussed above, the many clones generated during the immunization process are incompatible with the required specificity; therefore, monoclonal antibodies are generally necessary for such assays, and since they also need to have a reasonable affinity for the ligand, the development of a suitable pair of antibodies is really challenging. Therefore, it was a considerable advance when a high quality sandwich assay was finally developed by the company Mercodia [44], soon to be followed by other companies. Inherent in the sandwich technology is a lesser sensitivity to matrix effects. Moreover, with sandwich ELISA technology, another important problem was also solved: the volume requirements could also be minimized. This is essential because the traditional radioimmunoassays require larger volumes, 100 µL or more, particularly if extraction is applied, making it virtually impossible to measure glucagon concentrations in rodents, especially in mice [45]. This unfortunately means that most reported values obtained in mice with conventional techniques may not be accurate. The performance of the new sandwich ELISAs has been compared in a couple of studies using C-terminal radioimmunoassays (with extraction), showing very similar results. The Mercodia glucagon assay comes in two versions: A 10 µL version particularly suited for mouse studies and a 25 µL version for human use. The antisera are not the same, and to ensure enough sensitivity in the 10 µL assay, a pair of antibodies showing some cross-reaction with oxyntomodulin had to be used, resulting in a cross-reaction by oxyntomodulin in this assay of about 10% (personal communication). This may play a role in studies involving intestinal stimulation, whereas it may be suitable for studies not involving the GI tract. Otherwise, it is important to follow the instructions provided by the manufacturers. Failure to do so may result in untoward crossreactions and inaccurate measurements.

With the sandwich technology, it is of course also possible to measure other proglucagon products, and a sandwich ELISA for proglucagon 1–61 was recently described [46]. The secretion of PG 1–61 is particularly prominent during strong stimulation of the alpha cells, suggesting that it may act as an indicator of alpha cell stress. As mentioned, this component is particularly prevalent in patients with renal disease, but an explanation for this finding has not been found thus far [32].

Additionally, the gut derived peptides, oxyntomodulin and glicentin, may be measured accurately with the sandwich technique. Note that the first commercial oxyntomodulin assay (from Phoenix) was grossly inaccurate, yielding results which were about 1000-fold wrong [47]. Accurate oxyntomodulin assays are not commercially available yet to our knowledge [43].

## 5. Mass Spectrometry

Mass-spectrometry is, by some, called the ‘golden standard’. Mass-spectrometry analysis of small non-abundant peptides (e.g., glucagon) may be performed by several different approaches, each with limitations, sensitivity being the most obvious, but in fact also includes specificity. Why is that?

Bottom-up or shotgun mass-spectrometry is typically used to measure the plasma proteome and uses specific proteases (or none) to increase sensitivity [48]. The problem is the complexity of the proteome/peptidome also called the dynamic range. Albumin and immunoglobulins are the most abundant proteins, whereas glucagon is among some of the lowest abundant peptides. It is therefore necessary to get rid of the proteins to find the proglucagon peptides.

Four main factors contribute to the method’s ability to identify glucagon in a complex solution like plasma.

(1) Enrichment strategy by antibodies may suffer from specificity challenges (because the specificity then depends on the specificity of the applied antibodies), whereas chemical (solvent extraction) and physical-chemical isolation (high performance liquid chromatography or solid phase extraction) may cause insensitivity due to the inherent losses of the peptide of interest (recovery problems).

(2) Flow rate of the liquid chromatography, ‘number of precursor ions per time unit’, is important; by slowing this, one has a greater chance of identifying the peptide of interest.

(3) The acquisition of data (called data dependent and independent acquisitions) is crucial as this sets the ‘boarder’ from what gets analyzed and what that does not in the mass-spectrometry.

(4) The search strategy (search engine, including variables as stringency that affect the risk/chance of concluding that peptide A is endogenously present in the plasma and not generated during the sample preparation (e.g., during reduction/alkylation)). MaxQuant is the most applied tool worldwide but Peaks is also frequently used. Increasing the search frame (unbiased versus biased) is proportional to the ‘computer power’, and hence the time required. Therefore, searching for the (mass of the) glucagon sequence alone may lead to bias, as posttranslational modifications may change the molecular weight and hence the identification of the peptide.

Top-down or targeted proteomics (typically using a time-of flight instrument) had, until recently, limited sensitivity, but with the introduction of the TimsTOF by Bruker [49], this may change. In this setup, enrichment is not only mandatory for detection of peptides like glucagon in plasma but may also be a major limitation due to e.g., differential recovery of peptide a. This approach may together with selected reaction monitoring (SRM) constitute a strong tool for targeted mass-spectrometry but may suffer from the lack of specificity of the enrichment method used (e.g., antibodies against proglucagon) [50].

Collectively, mass-spectrometry is a powerful tool for evaluating the structure of a peptide or protein in plasma or tissue, but as the technology suffers from sensitivity challenges, it cannot be used to rule out or exclude the existence of e.g., glucagon in a given sample [51]. On the other hand, if the samples do contain glucagon-like peptides above the detection limits of the method, it is more accurate than an immunoassay to examine the heterogeneity/sequence of e.g., glucagon. As a method of quantification, on the other hand, mass spectrometry has its own problems.

## 6. How to Evaluate the Performance of a Glucagon Assay?

The usual criteria for assay validation may be applied: precision must be good; usually intra-assay coefficients of variation will be less than 10% (in our hands usually below 6%), but inter-assay variation may be a little higher (>10%). This means that it is important always to include quality controls and that samples that are to be compared should be analyzed together. Sensitivity must be high, allowing measurements down to 1 pmol/L. This is important, since the secretory performance of the alpha cells (the estimation of which is the purpose of all glucagon measurements) should not only be judged from their capacity to increase secretion in response to amino acids (the arginine test), but also show suppression by glucose [42]. Specificity towards the other proglucagon-derived peptides has been discussed above and is of course essential for the measurements. The specificity is probably best estimated by recovery experiments, where known amounts of the relevant peptides are added to the assay, but it may also be relevant to check in clinical experiments whether the assay performs as expected (e.g., showing suppression during hyperglycemica and great stimulation after arginine in healthy controls) [45]. Finally, there is the question of accuracy. Again, for this one, we may turn to recovery and dilution experiments, where known amounts of added glucagon must be recovered; even when added in small amounts to complex fluids such as plasma. Recovery studies using buffer as matrix are insufficient [43]. One way of testing accuracy is to compare performance against an accurate analysis. The question is whether an accurate analysis exists. Mass spectrometry has been proposed to represent the ultimate truth, but this is naturally not correct, since sample pretreatment may be associated with major and variable losses of analyte, while the sensitivity of the methods usually does not allow unbiased analysis as discussed above. Thus, mass spectrometry has its own sensitivity and accuracy problems.

Currently, there are important ongoing discussions regarding some of these issues, mainly focused on the question whether glucagon is indeed secreted from the gut or exclusively from the pancreas. In one study, glucagon was measured in pancreatectomized patients after oral glucose using both C-terminal radioimmunoassays, a sandwich ELISA and mass spectrometry (using physical-chemical enrichment strategy and an unbiased search frame), confirming paradoxical rises after oral glucose, in parallel with secretion of enteroglucagons and GLP-1 (illustrating the stimulation of the L-cell) [36]. These findings were in agreement with earlier reports on intestinal production of glucagon after total pancreatectomy in humans, as identified by C-terminal radioimmunoassay and size-exclusion chromatography [52]. This was interpreted to perhaps indicate aberrant processing of intestinal proglucagon in these patients, whose L-cells were more than usually stimulated because of the surgical reconstruction of the upper GI tact after the operation, in a manner resembling Roux-en-Y gastric bypass. In further studies, glucagon was also identified in intestinal extracts from these patients [53]. Another group looked at gastrectomized patients that had a similar reconstruction with overstimulation of the intestinal L-cells, and found secretion using the Mercodia assay (the version of the kit is not mentioned in the study), but not if a more thorough alternative washing protocol was applied [54]. They also used mass spectrometry to support their findings, and concluded that glucagon was not found in these samples. In a final study, the two protocols for the sandwich ELISA were thoroughly compared using clinical samples from Roux-en-Y patients and controls, and also included mass spectrometry analysis. In this study, results from mass spectrometry and the ELISA using the alternative protocol correlated well, whereas the correlation with results obtained with the traditional protocol was poor in RYGB operated patients. However, it was also clear that the alternative protocol was associated with systematically lower readings suggesting that analyte had been lost by this procedure. This was in particular the case for protein-induced secretion of glucagon which was completely lost [55].

From these considerations it is clear that in samples with potentially high values of alternative proglucagon products, great caution still needs to be exerted.

## 7. Recommendation for Measurement of Glucagon in Plasma

Preanalytical considerations, not presented in this paper, but covered elsewhere [56,57], are extremely important. To recap our recommendations:

- Use EDTA tubes for blood sampling and stored on ice until separation of plasma by temperature controlled (5 degrees Celsius) centrifugation. Always use low adsorption tubes.
- If a protease inhibitor is added (DPP-4, aprotinin, or similar), it is important to do this consistently across all samples—this seems to be most important for samples analyzed by ELISA, but the effect in the glucagon assays is not clear. It is not clear whether the use of P800 tubes is advantageous.
- Aprotinin or trasylol does not seem to be required when plasma is analyzed by C-terminal assays. Neprilysin inhibitors may increase circulating glucagon concentrations [58], but in EDTA plasma, neprilysin is inactive; thus, inhibitors do not need to be added.
- Storage of plasma samples in low-absorbent material at −20 degrees Celsius.
- Freeze-thaw maximum 1 time.
- Minimize hemolysis.
- If extraction/’cleaning’ is required, consider to use solvent and not solid phase extraction due to the poor recovery after solid phase extraction.

Analytical considerations:

- Check the recovery of both calibrator and purified glucagon peptide in the matrix intended for use (mouse or human plasma).
- Test if the precision of the analysis allows single or duplicate measurements (duplicate is generally advisable).
- Always follow the protocol by the manufacture.
- For ELISAs: consider the washing protocol and contact the vendor, but remember to focus on the aim of the study, e.g., if cross-reactivity to other proglucagon peptides is expected (e.g., after RYGB surgery), keep in mind you may lose the physiological dynamics regarding for example amino acid stimulated glucagon secretion due to the reduced recovery of native glucagon after intensified washing procedures.
- Classical C-terminal radioimmunoassay may be preferable for most purposes, but note that proglucagon 1-61 will crossreact (check renal function). However, if volume is critical, a validated sandwich ELISA is useful.

## 8. Conclusions

In this review, we have discussed the analytical challenges of measurement of plasma glucagon. We have, in particular, focused on strengths and limitations of radioimmunoassays, ELISAs, and mass-spectrometry based detection methods for measurement of glucagon. Finally, we have provided general guidelines for measurement of plasma glucagon that we hope may be helpful in the field on glucagon biology and guide critical assessment of commercially available glucagon assays.

## References

[1] Unger R.H. (1973). Radioimmunoassay of glucagon. Metabolism.

[2] Unger R.H., Eisentraut A.M., McCall M.S., Keller S., Lanzh C., Madison L.L. (1959). Glucagon antibodies and their use for immunoassay for glucagon. Proc. Soc. Exp. Biol. Med..

[3] Unger R.H., Eisentraut A.M., McCall R.H., Madison L.L. (1961). Glucagon antibodies and an immunoassay for glucagon. J. Clin. Invest..

[4] Unger R.H., Eisentraut A.M., McCall M.S., Madison L.L. (1962). Measurements of endogenous glucagon in plasma and the influence of blood glucose concentration upon its secretion. J. Clin. Invest..

[5] Unger R.H., Eisentraut A.M., Madison L.L. (1963). The effects of total starvation upon the levels of circulating glucagon and insulin in man. J. Clin. Invest..

[6] Ohneda A., Aguilar-Parada E., Eisentraut A.M., Unger R.H. (1969). Control of pancreatic glucagon secretion by glucose. Diabetes.

[7] McGavran M.H., Unger R.H., Recant L., Polk H.C., Kilo C., Levin M.E. (1966). A glucagon-secreting alpha-cell carcinoma of the pancreas. N. Engl. J. Med..

[8] Ohneda A., Parada E., Eisentraut A.M., Unger R.H. (1968). Characterization of response of circulating glucagon to intraduodenal and intravenous administration of amino acids. J. Clin. Invest..

[9] Muller W.A., Faloona G.R., Aguilar-Parada E., Unger R.H. (1970). Abnormal alpha-cell function in diabetes. Response to carbohydrate and protein ingestion. N. Engl. J. Med..

[10] Sasaki H., Rubalcava B., Baetens D., Blazquez E., Srikant C.B., Orci L., Unger R.H. (1975). Identification of glucagon in the gastrointestinal tract. J. Clin. Invest..

[11] Unger R.H., Ketterer H., Eisentraut A.M. (1966). Distribution of immunoassayable glucagon in gastrointestinal tissues. Metabolism.

[12] Valverde I., Rigopoulou D., Exton J., Ohneda A., Eisentraut A., Unger R.H. (1968). Demonstration and characterization of a second fraction of glucagon- like immunoreactivity in jejunal extracts. Am. J. Med. Sci..

[13] Valverde I., Rigopoulou D., Marco J., Faloona G.R., Unger R.H. (1970). Characterization of glucagon-like immunoreactivity (GLI). Diabetes.

[14] Murphy R.F., Buchanan K.D., Elmore D.T. (1973). Isolation of glucagon-like immunoreactivity of gut by affinity chromatography on anti-glucagon antibodies coupled to sepharose 4 B. Biochim. Biophys. Acta.

[15] Holst J.J. (1977). Extraction, gel filtration pattern, and receptor binding of porcine gastrointestinal glucagon-like immunoreactivity. Diabetologia.

[16] Heding L.G. (1971). Radioimmunological determination of pancreatic and gut glucagon in plasma. Diabetologia.

[17] Holst J.J., Aasted B. (1974). Production and evaluation of glucagon antibodies for radioimmunoassay. Acta Endocrinol..

[18] Ghatei M.A., Uttenthal L.O., Christofides N.D., Bryant M.G., Bloom S.R. (1983). Molecular forms of human enteroglucagon in tissue and plasma: Plasma responses to nutrient stimuli in health and in disorders of the upper gastrointestinal tract. J. Clin. Endocrinol. Metab..

[19] Holst J.J. (1980). Evidence that glicentin contains the entire sequence of glucagon. Biochem. J..

[20] Sundby F., Jacobsen H., Moody A.J. (1976). Purification and characterization of a protein from porcine gut with glucagon-like immunoreactivity. Horm. Metab. Res..

[21] Thim L., Moody A.J. (1981). The amino acid sequence of porcine glicentin. Peptides.

[22] Holst J.J. (1982). Evidence that enteroglucagon (II) is identical with the C-terminal sequence (residues 33–69) of glicentin. Biochem. J..

[23] Bataille D., Coudray A.M., Carlqvist M., Rosselin G., Mutt V. (1982). Isolation of glucagon-37 (bioactive enteroglucagon/oxyntomodulin) from porcine jejuno-ileum. Isolation of the peptide. FEBS Lett..

[24] Bataille D., Gespach C., Tatemoto K., Marie J., Coudray A., Rosselin G., Mutt V. (1981). Bioactive enteroglucagon (oxyntomodulin): Present knowledge on its chemical structure and its biological activities. Peptides.

[25] Moody A.J., Holst J.J., Thim L., Jensen S.L. (1981). Relationship of glicentin to proglucagon and glucagon in the porcine pancreas. Nature.

[26] Sheikh S.P., Baldissera F.G., Karlsen F.O., Holst J.J. (1985). Glicentin is present in the pig pancreas. FEBS Lett..

[27] Bell G.I., Santerre R.F., Mullenbach G.T. (1983). Hamster preproglucagon contains the sequence of glucagon and two related peptides. Nature.

[28] Bell G.I., Sanchez-Pescador R., Laybourn P.J., Najarian R.C. (1983). Exon duplication and divergence in the human preproglucagon gene. Nature.

[29] Orskov C., Holst J.J., Knuhtsen S., Baldissera F.G., Poulsen S.S., Nielsen O.V. (1986). Glucagon-like peptides GLP-1 and GLP-2, predicted products of the glucagon gene, are secreted separately from pig small intestine but not pancreas. Endocrinology.

[30] Mojsov S., Heinrich G., Wilson I.B., Ravazzola M., Orci L., Habener J.F. (1986). Preproglucagon gene expression in pancreas and intestine diversifies at the level of post-translational processing. J. Biol. Chem..

[31] Holst J.J., Bersani M., Johnsen A.H., Kofod H., Hartmann B., Orskov C. (1994). Proglucagon processing in porcine and human pancreas. J. Biol. Chem..

[32] Wewer Albrechtsen N.J., Kuhre R.E., Hornburg D., Jensen C.Z., Hornum M., Dirksen C., Svane M., Gasbjerg L.S., Jørgensen N.B., Gabe M.N. (2017). Circulating Glucagon 1–61 Regulates Blood Glucose by Increasing Insulin Secretion and Hepatic Glucose Production. Cell Rep..

[33] Jackson R.S., Creemers J.W., Farooqi I.S., Raffin-Sanson M.-L., Varro A., Dockray G.J., Holst J.J., Brubaker P.L., Corvol P., Polonsky K.S. (2003). Small-intestinal dysfunction accompanies the complex endocrinopathy of human proprotein convertase 1 deficiency. J. Clin. Invest..

[34] Baldissera F.G., Holst J.J. (1984). Glucagon-related peptides in the human gastrointestinal mucosa. Diabetologia.

[35] Holst J.J., Albrechtsen N.J.W., Gabe M.B.N., Rosenkilde M.M. (2018). Oxyntomodulin: Actions and role in diabetes. Peptides.

[36] Lund A., Bagger J.I., Wewer Albrechtsen N.J., Christensen M., Grøndahl M., Hartmann B., Mathiesen E.R., Hansen C.P., Storkholm J.H., van Hall G. (2016). Evidence of Extrapancreatic Glucagon Secretion in Man. Diabetes.

[37] Holst J.J., Bersani M., Conn P.M. (1991). Assays for peptide products of somatostatin gene expression. Methods in Neurosciences.

[38] Assan R., Slusher N. (1972). Structure-function and structure-immunoreactivity relationships of the glucagon molecule and related synthetic peptides. Diabetes.

[39] Heding L.G., Rasmussen S.M. (1972). Determination of pancreatic and gut glucagon-like immunoreactivity (GLI) in normal and diabetic subjects. Diabetologia.

[40] Holst J.J. (1997). Enteroglucagon. Annu. Rev. Physiol..

[41] Barnes A.J., Bloom S.R., Alford F.P., Russell R.C. (1976). Letter: Diabetes without glucagon. Lancet.

[42] Wewer Albrechtsen N.J., Veedfald S., Plamboeck A., Deacon C.F., Hartmann B., Knop F.K., Vilsboll T., Holst J.J. (2016). Inability of Some Commercial Assays to Measure Suppression of Glucagon Secretion. J. Diabetes Res..

[43] Bak M.J., Albrechtsen N.W., Pedersen J., Hartmann B., Christensen M., Vilsbøll T., Knop F.K., Deacon C.F., Dragsted L.O., Holst J.J. (2014). Specificity and sensitivity of commercially available assays for glucagon and oxyntomodulin measurement in humans. Eur. J. Endocrinol..

[44] Wewer Albrechtsen N.J., Hartmann B., Veedfald S., Windelov J.A., Plamboeck A., Bojsen-Moller K.N., Idorn T., Feldt-Rasmussen B., Knop F.K., Vilsbøll T. (2014). Hyperglucagonaemia analysed by glucagon sandwich ELISA: Nonspecific interference or truly elevated levels?. Diabetologia.

[45] Wewer Albrechtsen N.J., Kuhre R.E., Windelov J.A., Orgaard A., Deacon C.F., Kissow H., Hartmann B., Holst J.J. (2016). Dynamics of glucagon secretion in mice and rats revealed using a validated sandwich ELISA for small sample volumes. Am. J. Physiol. Endocrinol. Metab..

[46] Wewer Albrechtsen N.J., Asmar A., Jensen F., Torang S., Simonsen L., Kuhre R.E., Asmar M., Veedfald S., Plamboeck A., Knop F.K. (2017). A sandwich ELISA for measurement of the primary glucagon-like peptide-1 metabolite. Am. J. Physiol. Endocrinol. Metab..

[47] Laferrere B., Swerdlow N., Bawa B., Arias S., Bose M., Olivan B., Teixeira J., McGinty J., Rother K.I. (2010). Rise of oxyntomodulin in response to oral glucose after gastric bypass surgery in patients with type 2 diabetes. J. Clin. Endocrinol. Metab..

[48] Aebersold R., Mann M. (2016). Mass-spectrometric exploration of proteome structure and function. Nature.

[49] Meier F., Brunner A.D., Koch S., Koch H., Lubeck M., Krause M., Goedecke N., Decker J., Kosinski T., Park M.A. (2018). Online Parallel Accumulation-Serial Fragmentation (PASEF) with a Novel Trapped Ion Mobility Mass Spectrometer. Mol. Cell Proteom..

[50] Lee A.Y., Chappell D.L., Bak M.J., Judo M., Liang L., Churakova T., Ayanoglu G., Castro-Perez J., Zhou H., Previs S. (2016). Multiplexed Quantification of Proglucagon-Derived Peptides by Immunoaffinity Enrichment and Tandem Mass Spectrometry after a Meal Tolerance Test. Clin. Chem..

[51] Albrechtsen N.J.W. (2017). Measurement of Gastrointestinal Hormones. Dan. Med. J..

[52] Holst J.J., Pedersen J.H., Baldissera F., Stadil F. (1983). Circulating glucagon after total pancreatectomy in man. Diabetologia.

[53] Jorsal T., Wewer Albrechtsen N.J., Christensen M.M., Wandall E., Langholz E., Friis S., Worm D., Ørskov C., Støving R.K., Andries A. (2019). Investigating intestinal glucagon after Roux-en-Y gastric bypass surgery. J. Clin. Endocrinol. Metab..

[54] Roberts G.P., Kay R.G., Howard J., Hardwick R.H., Reimann F., Gribble F.M. (2018). Gastrectomy with Roux-en-Y reconstruction as a lean model of bariatric surgery. Surg. Obes. Relat. Dis..

[55] Alexiadou K., Cuenco-Shillito J.H.J., Wewer Albrechtsen N.J., Ilesanmi I., Kamocka A., Tyharakan G. (2019). Glucagon secretion profiles in type 2 diabetes before and after bariatric surgery: One-year prospective study.

[56] Wewer Albrechtsen N.J., Bak M.J., Hartmann B., Christensen L.W., Kuhre R.E., Deacon C.F., Holst J.J. (2015). Stability of glucagon-like peptide 1 and glucagon in human plasma. Endocr. Connect..

[57] Cegla J., Jones B.J., Howard J., Kay R., Creaser C.S., Bloom S.R., Tan T.M. (2017). The preanalytical stability of glucagon as measured by liquid chromatography tandem mass spectrometry and two commercially available immunoassays. Ann. Clin. Biochem..

[58] Wewer Albrechtsen N.J., Mark P.D., Terzic D., Hansen L.H., Andersen U.O., Hartmann B., Carr R.D., Gustafsson F., Deacon C.F., Holst J.J. (2019). Sacubitril/valsartan augments postprandial plasma concentrations of active GLP-1 when combined with sitagliptin in men. J. Clin. Endocrinol. Metab..

